# Evolution of Artificial Intelligence in Medical Education From 2000 to 2024: Bibliometric Analysis

**DOI:** 10.2196/63775

**Published:** 2025-01-30

**Authors:** Rui Li, Tong Wu

**Affiliations:** 1 Emergency Department, Tongji Hospital, Tongji Medical College, Huazhong University of Science and Technology Wuhan China; 2 Department of Obstetrics and Gynecology Tongji Hospital, Tongji Medical College Huazhong University of Science and Technology Wuhan China

**Keywords:** artificial intelligence, medical education, bibliometric, citation trends, academic pattern, VOSviewer, Citespace, AI

## Abstract

**Background:**

Incorporating artificial intelligence (AI) into medical education has gained significant attention for its potential to enhance teaching and learning outcomes. However, it lacks a comprehensive study depicting the academic performance and status of AI in the medical education domain.

**Objective:**

This study aims to analyze the social patterns, productive contributors, knowledge structure, and clusters since the 21st century.

**Methods:**

Documents were retrieved from the Web of Science Core Collection database from 2000 to 2024. VOSviewer, Incites, and Citespace were used to analyze the bibliometric metrics, which were categorized by country, institution, authors, journals, and keywords. The variables analyzed encompassed counts, citations, H-index, impact factor, and collaboration metrics.

**Results:**

Altogether, 7534 publications were initially retrieved and 2775 were included for analysis. The annual count and citation of papers exhibited exponential trends since 2018. The United States emerged as the lead contributor due to its high productivity and recognition levels. Stanford University, Johns Hopkins University, National University of Singapore, Mayo Clinic, University of Arizona, and University of Toronto were representative institutions in their respective fields. *Cureus*, *JMIR Medical Education*, *Medical Teacher*, and *BMC Medical Education* ranked as the top four most productive journals. The resulting heat map highlighted several high-frequency keywords, including performance, education, AI, and model. The citation burst time of terms revealed that AI technologies shifted from imaging processing (2000), augmented reality (2013), and virtual reality (2016) to decision-making (2020) and model (2021). Keywords such as mortality and robotic surgery persisted into 2023, suggesting the ongoing recognition and interest in these areas.

**Conclusions:**

This study provides valuable insights and guidance for researchers who are interested in educational technology, as well as recommendations for pioneering institutions and journal submissions. Along with the rapid growth of AI, medical education is expected to gain much more benefits.

## Introduction

Artificial intelligence (AI) seeks to develop innovative machines that are capable of responding in ways similar to human intelligence. Its scope encompasses robotics, language recognition, image recognition, natural language processing, and expert systems. In recent years, the integration of AI into medical education (AIME) has significantly transformed traditional teaching and learning methodologies [1,2]. It can enhance clinical reasoning training, facilitate adaptive learning, construct innovative medical teaching platforms, and facilitate critical care simulation teaching. For instance, the AI-powered simulation mannequins called SimMan 3G PLUS allows medical students to practice their clinical skills and teamwork in a fully controlled and immersive environment. Augmented reality and virtual reality technologies enable students to gain valuable hands-on experience by recreating real-life situations, without compromising patient safety [3]. In other cases, AI helps tailor educational content based on individual students’ needs and abilities, and it not only fosters academic success but also cultivates critical thinking [1,4]. AI applications have become an important tool and situation for both educators and students.

As AI continues to evolve and advance, its role in promoting medical education is expected to grow. Despite many publications that have explored AI’s application in medical research, they are limited to specific clinical questions in cardiology, dentistry, oncology, etc [5,6]. No bibliometric analysis has ever been conducted to assess medical education, which is essential for medical talent reserve. Therefore, a comprehensive analysis of current scientific literature is imperative for the continued advancement of AIME.

Bibliometrics uses mathematical and statistical methodologies to evaluate scholarly productivity and publication patterns within a specific discipline. We used bibliometrics to analyze the contemporary academic landscape, research priorities, and emerging trends in AIME in the 21st century. We revealed the knowledge clusters, scientific social patterns, and evolutionary nuances from an objective standpoint. This work not only simplifies navigation for researchers across disciplines but also gives valuable guidance to newcomers in the field, directing them toward promising avenues for future research.

## Methods

### Study Design

The research adhered to the step-by-step guidelines of bibliometric analysis and followed the reporting guideline of bibliometric reviews in biomedical literature [7].

### Data Collection

The Web of Science Core Collection database was selected as the primary tool to identify relevant publications for this study. This database is well-known for its exceptional bibliometric research capabilities across over 190 subject areas and offers manual-checked literature retrieval. A 2-step process was used to construct an effective retrieval strategy. First, we extracted search terms from relevant systematic reviews and meta-analyses. Second, these search terms were meticulously reviewed and refined by authors (TW and RL) to ensure adequate coverage of all relevant research topics. The final search strategy was formulated based on this collaborative assessment: TS=(“artificial intelligence” or AI or “convolutional neural network” or “recurrent neural network” or “long short-term neural memory” or “machine learning” or “genetic algorithm” or “evolutionary algorithm” or “artificial neural network” or “support vector machine” or “fuzzy logic” or “random forest” or “deep learning” or “natural language processing” or “speech recognition” or “computer vision” or “smart robot” or “video recognition” or “image recognition”) and TS=(medical education or medicine education). Data collection took place on October 1, 2024, with publication years restricted from 2000 to 2024. No language restrictions were applied during the search process. Full records of retrieved papers were exported in plain text format.

### Selection of Eligible Studies

Since search engines may yield results that do not fully align with the intended criteria, it is necessary to perform a manual screening process to identify the final included literature. To address this, two authors (TW and RL) independently screened the titles and abstracts of available studies based on the following exclusion criteria: (1) publications unrelated to AI. For example, some topics are associated with artificial insemination. (2) Publications unrelated to medical education, such as engineering curriculum and patient education. Notably, a majority of these studies have applied AI technologies to clinical questions and research, and only a small number pertained to medical education. Any discrepancies between the studies selected by the authors were resolved through a consensus meeting to reach a binding decision.

### Data Cleaning and Preprocessing

The records from the Web of Science Core Collection database undergo rigorous selection criteria adhere to meticulous curation standards, and it partly enhances our research quality [8]. To further ensure accuracy and consistency, we performed a thorough data cleaning process. This involved the elimination of duplicate papers, which were identified through digital objective identifiers and study titles. The biblioshiny package in RStudio was used to standardize the names of authors and institutions. Any variations in author names were consolidated. As an author usually affiliates with institutions at different times, only the institution of the author at the time of publication is retained. Synonyms, aliases, and singular or plural forms, such as “AR,” “augmented reality,” and “augmented realities” were cleaned using a thesaurus file. The data cleaning was conducted manually by two authors (TW and RL).

### Data Analysis and Visualization

To present the knowledge structure and emerging research trends, VOSviewer (version 1.6.18; Leiden University), Incites (Clarivate), InteractiVenn (Universidade de São Paulo), and Citespace (Drexel University) were used. Incites is a research evaluation tool developed by Clarivate Analytics, and the scores of citation impact, H-index, journal impact factor, international collaborations, study influence, and immediacy index were directly obtained in Incites. The citation impact is calculated by dividing the citation impact by the number of years since publication. The H-index indicates that H papers published by an author have been cited at least H times, thereby serving as a gauge of both scholarly productivity and influence. The journal impact factor was used based on the 2023 Journal Citation Reports. International collaborations were assessed to reveal the extent of interdisciplinary cooperation among coauthors hailing from various nations, highlighting the global reach and collaborative nature of the research topic. The study’s influence measures the mean influence of a study within the first 5 years of post publication, providing a view of its long-term impact. Concurrently, the immediacy index represents the average frequency with which a study is cited in the year of its publication, offering insight into the immediate reception in the academic community. The VOSviewer software was used to cluster countries, institutions, journals, and keywords. The Citespace software was used to identify keyword clusters and citation burst time. The specific parameter settings for analysis in VOSviewer and Citespace are provided in Tables 1 and 2. The latent semantic indexing and log-likelihood ratio algorithms were used for literature clustering. The InteractiVenn tool was used to identify the specific and overlapping journals in 4 categories: count, citation, top 10% papers, and cooperative work. To compare the frequencies of keywords in the AI and medical education fields, the keywords were classified based on their basic definition, and lists of professional terms or vocabulary or terminology.

**Table 1:** Information for clustering using the VOSviewer.

Item	Type	Algorithm	Normalization method	Minimum document or occurrence^a^
Country	Coauthorship	Majorization	Full counting	12
Organization	Coauthorship	Majorization	Full counting	15
Journal	Citation	Majorization	—^b^	12
Keyword	Co-occurrence	Majorization	Full counting	33

^a^We set no limit on the minimum citation.

^b^Not applicable.

**Table 2:** Information for keyword cluster using the Citespace.

Setting	Value
Time slicing	From January 2000 to October 2024, one year per slice
Text processing	Title, abstract, author keywords, and keywords plus
Node type	Keywords
Links	Strength: cosinel Scope: within slices
Selection criteria	k=7

### Ethical Considerations

The institutional review board of Tongji Hospital deemed ethical approval unnecessary for this study.

## Results

### Baseline Characteristics

**Figure 1:**
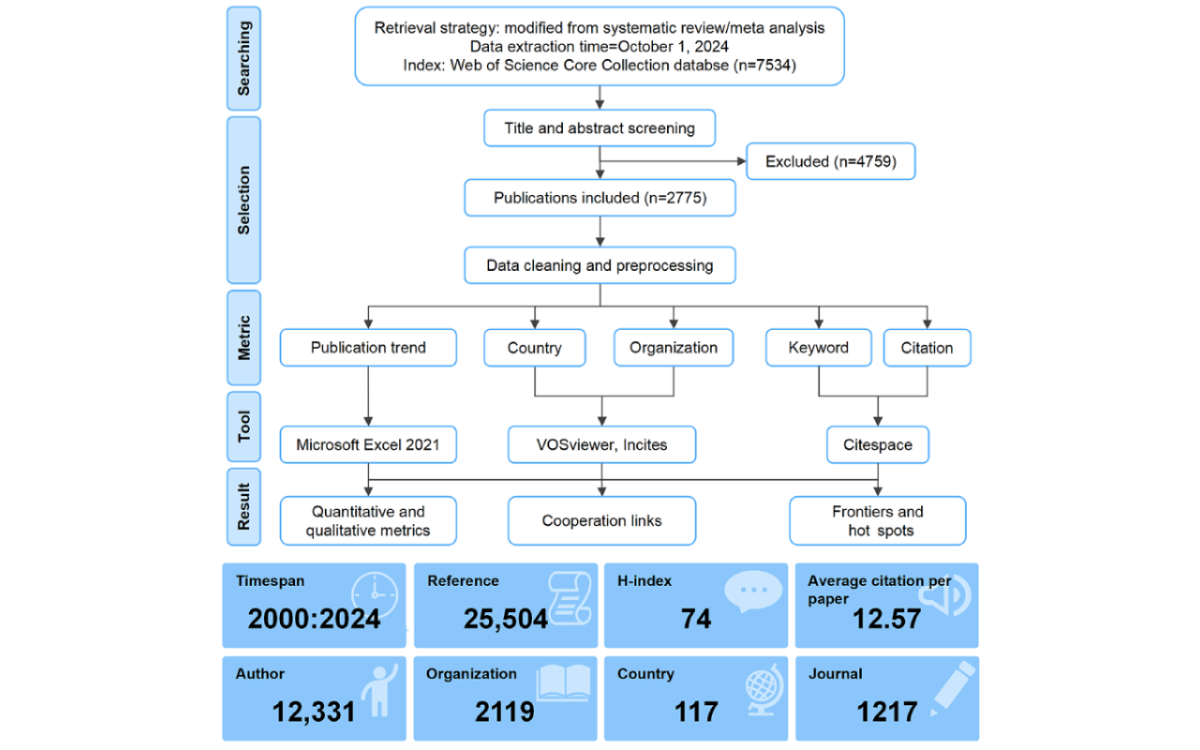
Flowchart and basic information of retrieval strategy, screen process, and analysis methods.

**Figure 2:**
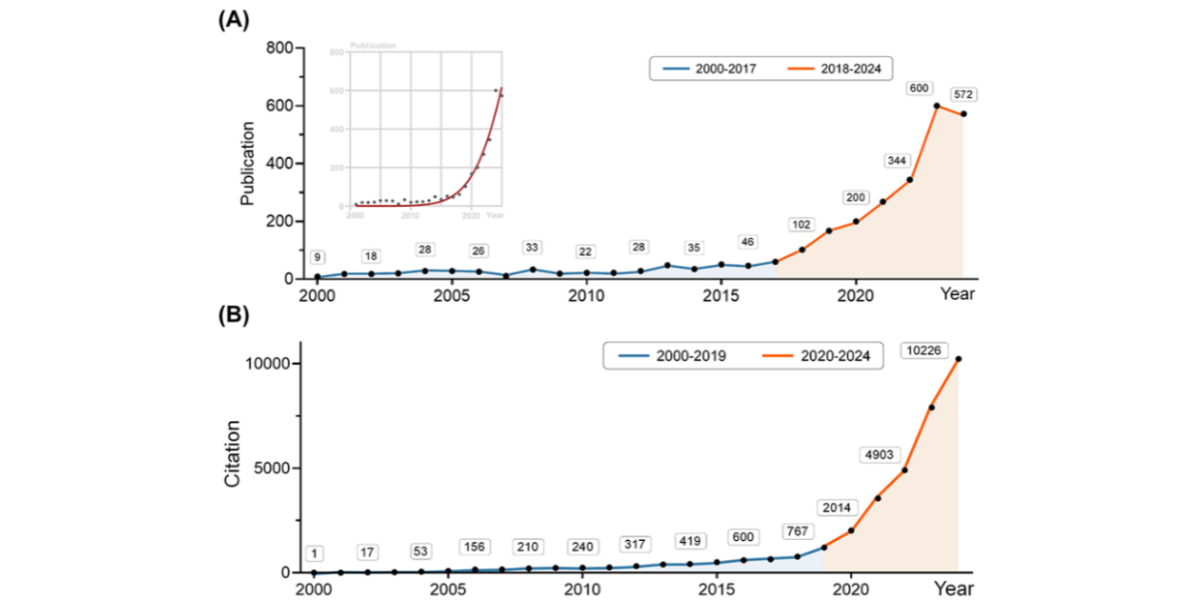
Baseline bibliometric characteristics of all eligible documents. (A,B) Line chart showing the annual number of documents and citations.

This study initially retrieved a total of 7534 publications from the search. Figure 1 provides a detailed flowchart illustrating the publication retrieval and selection process. After title and abstract screening, 2775 publications remained for further bibliometric analysis. The academic publications underwent a relatively flat growth in publication numbers from 2000 to 2017 (Figure 2A). However, the number of annual papers exhibited exponential growth since 2018, indicating the flourished development in this field. The citation counts also demonstrated a consistent rise, surpassing 10,000 citations in 2024 (Figure 2B). These documents encompassed original studies (n=1769, 63.75%), proceeding papers (n=467, 16.83%), reviews (n=237, 8.54%), and meeting abstracts (n=67, 2.41%). Proceeding papers typically contain the latest research findings and methods, as well as techniques within a scientific field, and their high percentage indicates that AIME is receiving much attention and developing rapidly. The research areas are primarily focused on Education Scientific Disciplines (n=415), Medicine General Internal (n=334), Health Care Sciences Services (n=323), Engineering Biomedical (n=264), and Surgery (n=255). It suggested that AIME is an interdisciplinary topic that needs support from specialized educators, clinical doctors, hospital administrators, and engineers.

### Productive and Influential Countries

**Figure 3:**
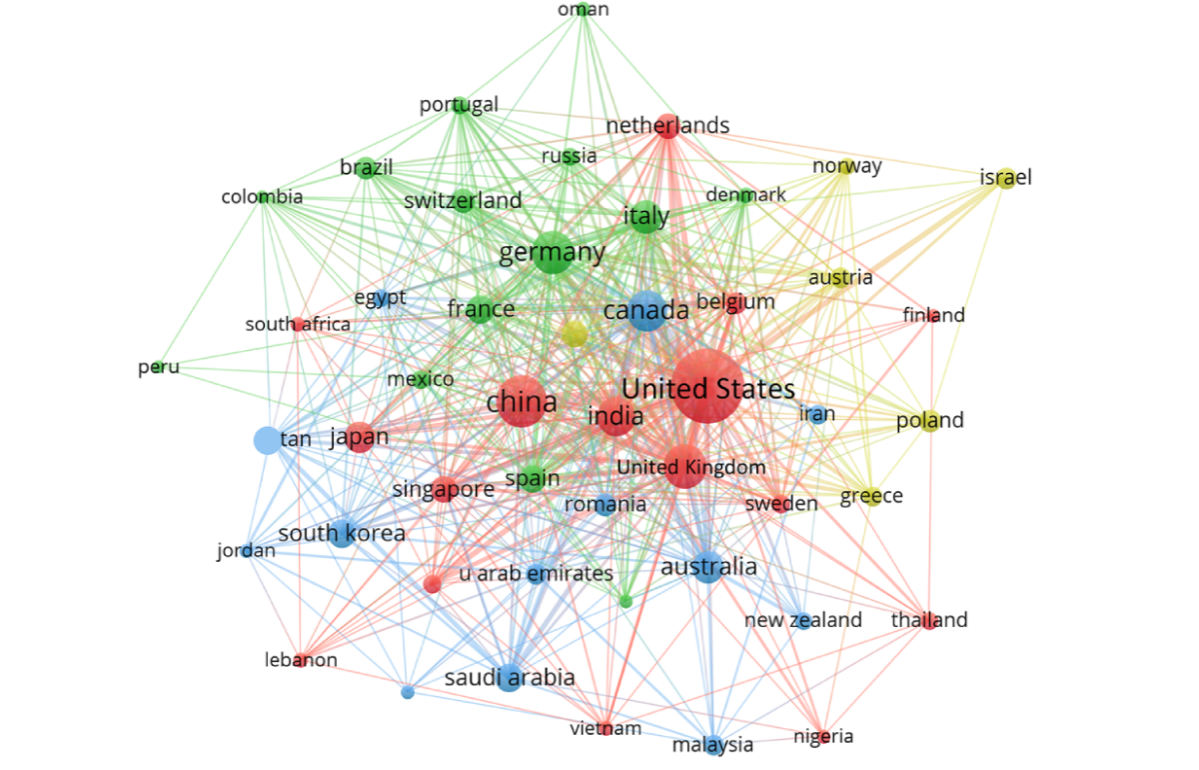
Relationship and cluster of countries. Cooperative relationships networks among countries.

In terms of national performance, 117 countries participated in the global discourse. The majority of research publications originated from Europe, North America, and Asia. The United States emerged as the primary contributor due to its high productivity (n=851 publications) and recognition levels (n=11,598 citations), exhibiting both the highest international and domestic collaborations. Although Chinese researchers published more papers than scholars from the United Kingdom (274 vs 227), their citations were relatively lower (3302 vs 3823). It may possibly be due to the limited innovation, restricted dissemination, and language barriers. The national cluster map revealed a diverse regional distribution pattern facilitated by these collaborative relationships (Figure 3). There was a strong collaboration among numerous countries, particularly close ties between the United States, China, the United Kingdom, and India.

### Institutional Performance

**Figure 4:**
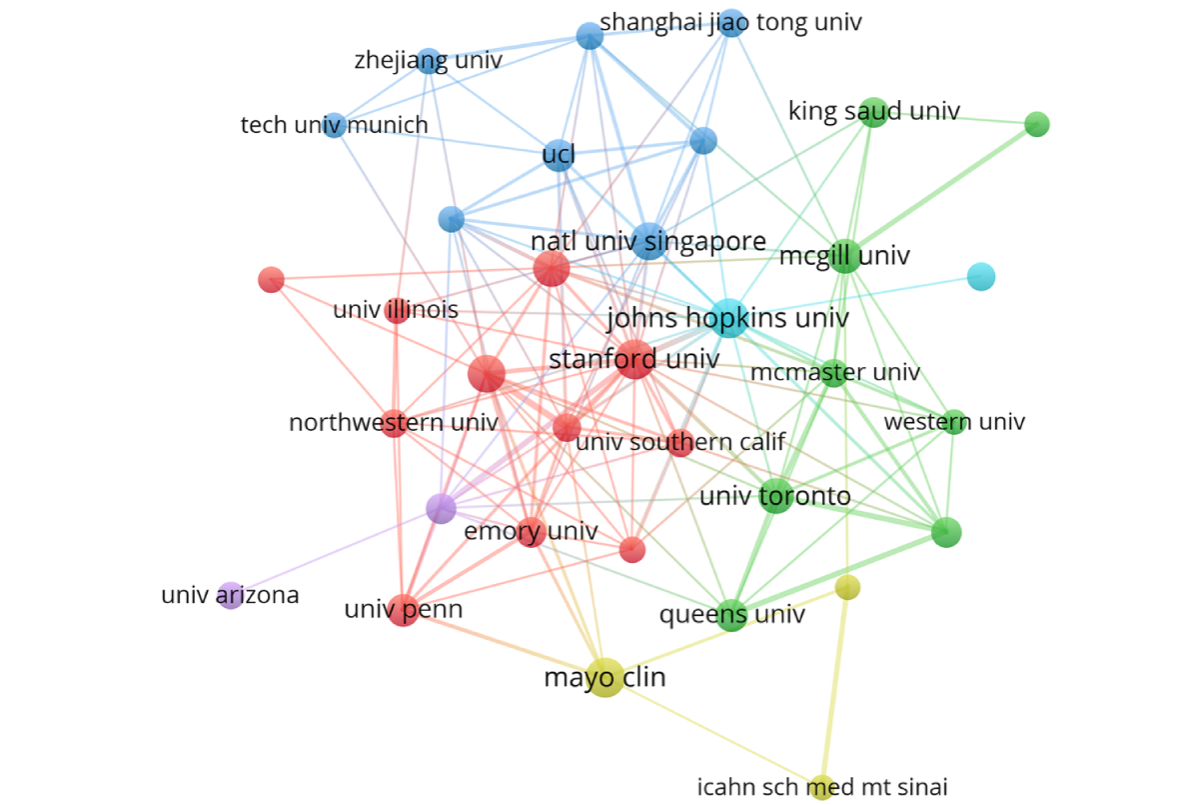
Visualization of institutional cooperation. Cooperative relationships networks.

Altogether, 2119 institutions have participated in AIME studies. The top 10 institutions publishing the most papers are presented in Table 3. Among these, 4 institutions have published over 50 papers. Specifically, Harvard University emerged as the most prolific institution (n=70), and established the most international collaboration (n=35), while the University of London had the highest citation impact (n=27). The collaborative connections showed six clusters of countries publishing more than 40 papers (Figure 4). Stanford University, Johns Hopkins University, National University of Singapore, Mayo Clinic, University of Arizona, and University of Toronto were representative institutions in their respective cluster. Interestingly, regarding the impact relative to the world, the Alibaba Group had the highest score (value=61.43), followed by SUNY Downstate Health Sciences University (value=32.17), and Bukovinian State Medical University (value=25.14).

**Table 3:** Top 10 productive institutions of artificial intelligence in the medical education field.

Institution	Document	Citation	Citation Impact	International collaboration	H-Index	Impact relative to world
Harvard University	70	1085	15.50	35	17	1.25
University of California System	60	861	14.35	17	16	1.16
University of London	52	1211	23.29	27	17	1.88
Johns Hopkins University	51	1003	19.67	21	17	1.59
Stanford University	48	834	17.38	22	16	1.40
Harvard Medical School	47	588	12.51	25	13	1.01
Mayo Clinic	44	401	9.11	12	10	0.74
National University of Singapore	43	433	10.07	24	12	0.81
University of Toronto	39	624	16.00	14	14	1.29
University of Michigan	39	308	7.90	19	9	0.64

### Participants of the Source Journals

**Figure 5:**
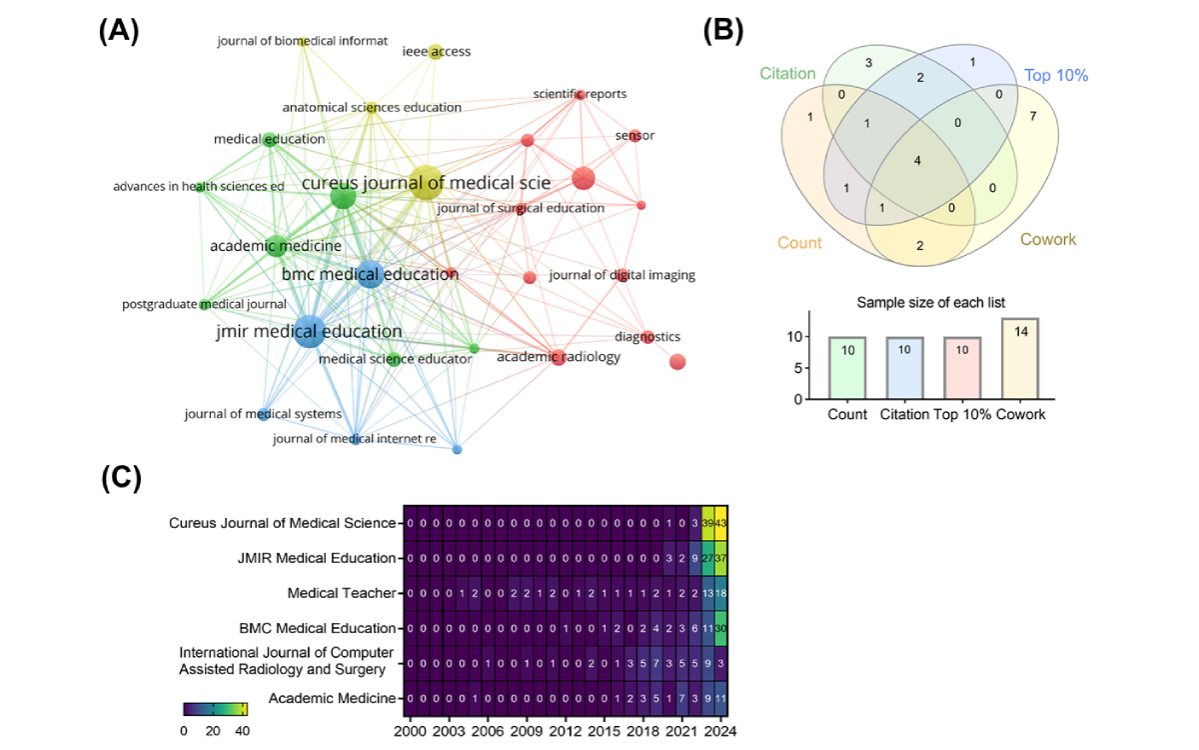
Participated journals concerning artificial intelligence in the medical education field. (A) Relationship of representative journals. (B) Three significant journals were identified through a Venn diagram analysis of top publication volume, citation, top 10%, and cowork. (C) Annual academic output for the top five journals.

*Cureus* (n=78), *JMIR Medical Education* (n=72), *Medical Teacher* (n=54), and *BMC Medical Education* (n=54) ranked as the top 4 most productive journals among the 1217 involved journals (Figure 5A and Table 4). *JMIR Medical Education* published the most high-impact papers (n=48), and the second one, Cureus, only published 26. It is reasonable as *JMIR Medical Education* lies in the Q1 quartile, and likely receives more high-quality submissions, whereas Cureus is in the Q3 quartile. Cureus, *JMIR Medical Education*, *Medical Teacher*, and *International Journal of Computer Assisted Radiology and Surgery* were overlapped using a Venn diagram (Figure 5B). It was worth highlighting that Cureus and *JMIR Medical Education* had just begun to publish AIME-associated papers in 2020 (Figure 5C). Their open access characteristic helped them receive much attention in recent years. The publishers were diverse among the top 10 publication sources. More than half of these journals (7/10) published top 10% papers over 10. Notably, a significant proportion of the journals (n=1460, 52.61%) offered open access publishing, with gold access accounting for 33.26% (n=923) of this share.

**Table 4:** Top five journals ranked by the counts of publications.

	*Cureus*	*JMIR Medical Education*	*Medical Teacher*	*BMC Medical Education*	*International Journal of Computer-Assisted Radiology And Surgery*
Document, n	78	72	54	54	46
Citation, n	910	751	1139	494	733
Top 10% paper	26	48	24	22	8
Publisher	Springer Nature	JMIR Publications Inc	Taylor & Francis Ltd	BMC	Springer Heidelberg
Cited half-life	2.5	2.1	9.1	4.4	4.8
Journal impact factor	1.1	3.2	4.7	3.4	3.1
Study influence	0.26	—^a^	1.53	0.86	0.74
Immediacy index	0.2	8.7	0.9	0.4	0.4
Open access	100%	97.22%	96.3%	24.07%	36.96%
JCI rank	173/329	18/85	10/85	15/175	67/204
JCI quartile	Q3	Q1	Q1	Q1	Q2

^a^Not applicable.

### Keywords of Research Hot Spots

**Figure 6:**
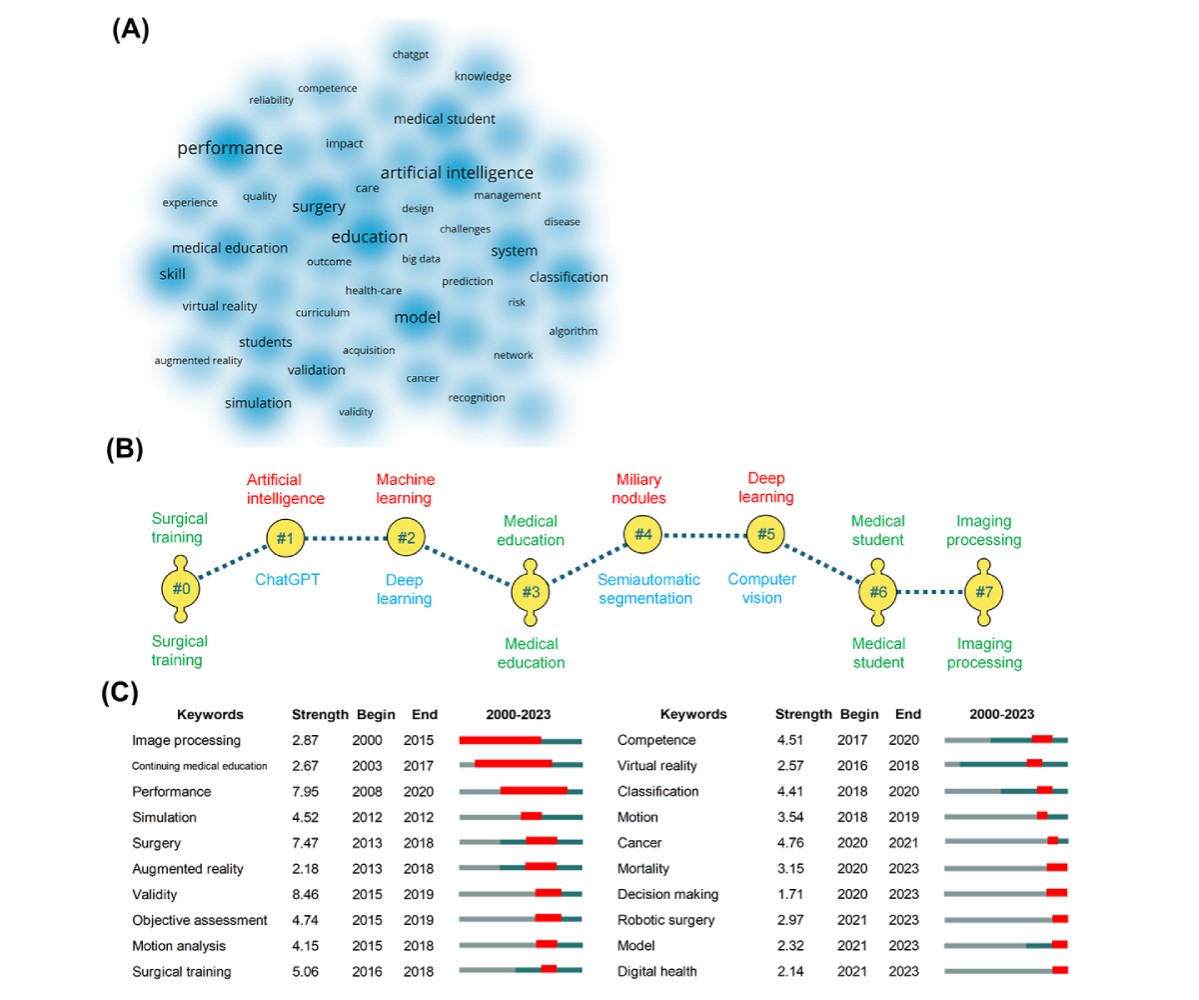
Keywords showing the hotspots and clusters. (A) Top keywords in the AIME field. (B) Eight clusters were obtained and unfolded using the latent semantic indexing (red), log-likelihood ratio (black) algorithms, or shared by both (green). (C) Keyword burstness panel showing the strength and duration. AIME: artificial intelligence into medical education.

The study identified 7773 keywords from the titles and abstracts of the research materials, reflecting the central themes, areas of interest, and potential future developments within the discipline. The heat map highlighted high-frequency keywords, including performance (n=139), education (n=123), artificial intelligence (n=118), and model (n=103; Figure 6A). We next classified keywords according to the AI and medical education associated categories. The model (n=103), system (n=82), virtual reality (n=51), recognition (n=29), and algorithm (n=27) were popular topics, while most education focus was put on surgery (n=100), skill (n=90), simulation (n=75), classification (n=68), and diagnosis (n=47). The subsequent analysis produced eight distinct keyword clusters using log-likelihood ratio and latent semantic indexing algorithms (Figure 6B). Both algorithms identified #0 surgical training, #3 medical education, #6 medical student, and #7 imaging processing as hub keywords. It suggested the critical role of AI applications in surgical operations and clinical image information. Citespace burst detection could reflect the research trends and innovative directions (Figure 6C). Notably, the citation burst time of terms revealed that AI technologies shifted from imaging processing (2000), augmented reality (2013), and virtual reality (2016) to decision-making (2020) and model (2021). Keywords such as mortality and robotic surgery persisted into 2023, suggesting the ongoing recognition and interest in these areas. Digital health involves the use of health apps, wearable equipment, and communication tools, which have been incorporated with AI. These devices not only provide personalized educational experiences and support mental health but also play a significant role in clinical teaching, disease prevention, and health promotion.

## Discussion

### Principal Findings

AIME publication exhibits exponential growth these years, from approximately 50 counts in 2017 to 600 in 2023. This surge is further bolstered by the development of generative AI tools, and related national-level policies support. Here, we discuss the applications of AIME according to the keywords.

### Machine Learning

Machine learning (ML), a subset of AI, is a technology that autonomously discerns inner patterns and relationships without explicit human instructions. For instance, after analyzing a large collection of cat and dog images, ML can identify distinguishing features, and subsequently differentiate between a cat and a dog in new photographs. The growing prominence of ML in medical education is evidenced by the surge in related topics, the approval of ML-based products, and the proliferation of entrepreneurial initiatives. ML-centric technologies have already been applied in clinical decision support systems, teaching tools, simulation, and training. An ML-based clinical decision support system could effectively integrate general information about HIV patients, including demographics, medical history, CD4+ lymphocyte count, viral load, genotypic data, and treatment history, to recommend an optimal combination antiretroviral therapy [9]. More personalized advice on the appropriate dosage or duration of treatment will be provided with the help of other advanced ML algorithms [10]. In this context, it is vital to educate the next generation of medical professionals with adequate ML knowledge, enabling them to incorporate the outputs of ML tools into clinical decision-making, becoming part of this emerging data science revolution [11]. In the future, inexperienced medical students will use evidence-based learning models, like IBM’s Watson Oncology system, as ordinary tools to interpret clinical data, make informed decisions, and recommend cancer treatments, in highly accordance with multidisciplinary teams [12].

Medical students require educational feedback to understand their performance and identify areas for improvement [13]. Traditional educational evaluation after surgical training falls short of providing timely, adequate, and objective assessment. A human rater is usually required to observe the video review to give a written or oral evaluation. The feedback is subjective and time-consuming, as well as limited to visual observation. With the help of automated ML-based AI assessments, the surgeon’s performance can be objectively captured in a less resource-intensive way than human grading [14]. The motion, force, and vibration of robotic instruments are recorded according to the recognized structural metrics, like the Global Evaluative Assessment of Robotic Skill or the Objective Structured Assessment of Technical Skill [15].

### Deep Learning

Deep learning (DL), a subset of ML, is often used to tackle complex tasks such as visual recognition, speech recognition, and natural language processing. This is achieved through the use of advanced architectures like convolutional neural networks, deep belief networks, and stacked auto-encoder networks. DL has been applied across medical undergraduate education, postgraduation education, and continuing education. A notable example is its use during medical retina rotations, where residents may not be fully trained due to limited time or access to complete their learning objectives. To address this, a DL-based model was developed to analyze a vast collection of retina images sourced from three public datasets, subsequently creating a comprehensive dataset for residents. These images were then distributed to each resident to aid in diagnosis, differential diagnosis, and therapeutic planning. The allocation system is tailored to each resident’s case history, academic level, and performance, ensuring that those struggling with specific cases receive additional exposure to similar retinal conditions [16]. This approach enables AI models to identify the residents who will derive the most benefit from particular clinical cases, thereby significantly enhancing individualized ophthalmology education.

Additionally, DL technologies have been used to predict the difficulty of medical licensing examination questions, promoting more accurate assessments of examination difficulty. However, although DL models can effectively differentiate between cats and dogs, they do so by analyzing potentially hundreds or thousands of variables. The complexity and opacity of these variables often render them incomprehensible to humans. Consequently, there is an urgent need for improved interpretability methods in future DL applications to enhance understanding and transparency.

### Natural Language Processing

Natural language processing plays a crucial role in smart health because of its ability to analyze and comprehend human language. The ChatGPT, developed by OpenAI Corporation, serves as a monumental tool for the natural language processing application. As for AIME, GPT-4 has been used for teaching cases, student analysis, creative writing, personalized learning guidance, and psychological support. ChatGPT is demonstrated to be effective in generating surgical procedure summaries, and its performance cannot be distinguished from a board-certified surgeon by less experienced residents [17]. In other cases, ChatGPT is used to teach the skills of breaking bad news [18], reasoning-based multiple-choice questions [19], and qualified examinations, including the Situational Judgement Test for final-year medical students in the United Kingdom [20]. These capabilities have revolutionized medical education and contributed to the overall improvement of health care delivery.

### Segmentation

Image segmentation is a crucial AI technology in the radiology and pathology fields to accurately identify and delineate regions of interest, such as tumor lesions, ischemic tissues, and subcellular structures [21]. Traditional manual segmentation is not only time-consuming for physicians to learn and practice but also results in measurement variability that heavily depends on the observer’s experience. In contrast, computer-assisted segmentation methods reduce the subjectivity and variability inherent in manual approaches, decrease processing time, and require minimal training. The use of image segmentation algorithms has led to significant improvements in the sensitivity and efficiency of detecting pulmonary nodules. Furthermore, these algorithms have been shown to enhance learning interest, bolster self-directed learning capabilities, sharpen problem-solving skills, and foster innovative thinking among medical trainees [22].

Effective surgical education for young surgeons presents significant challenges in practice. A segmentation-based system can identify key anatomical structures such as arteries, lymph nodes, and nerves during rectal cancer surgeries. Studies have shown the positive educational impact of AI-assisted videos in surgical training [23]. Some other AI technologies can facilitate surgical navigation and detect adverse events [24]. Taken together, real-time object segmentation is expected to play a major role in surgical education.

### Comparison to Prior Work

AI has been increasingly used to enhance diagnostic accuracy, improve patient care, and facilitate the development of new treatments across various medical fields. In cardiology, for example, AI-powered algorithms are used to analyze electrocardiograms and detect arrhythmias, while in dentistry, AI-assisted diagnostic tools have been developed to identify oral pathologies [25,26]. Similarly, in oncology, AI-driven platforms are used to predict tumor growth and response to treatment [27]. Rather than concentrating on specific clinical questions, this study adopts a broader perspective to investigate the role of AI in medical education as a whole. High-quality medical education is the key to improving the level of medical care. Through our bibliometric analysis, research trends and hot topics in this field were identified, offering insights into how AI is transforming medical education globally.

### Future Direction

AIME has witnessed a remarkable evolution from the initial enthusiasm phase to the current acceptance situation [2]. Although the potential of AI technologies is expected to revolutionize medical education, related ethical considerations and challenges should be carefully examined. One major concern is whether AI might diminish the competence of medical students by increasing their reliance on external tools. Additionally, there is apprehension about the possibility of AI completely replacing medical educators. Issues such as bias, hallucinations, and uncertainties have further contributed to hesitancy in the acceptance and practical application of AI in the medical field [28]. It was necessary for a balanced approach to ensure sustainable implementation and find practical ways to incorporate AI into curricula. As deeper investigation is conducted, AI will be an integral part of medical education, highlighting a journey of personal and professional growth alongside technological adoption.

Since the release of ChatGPT, AI-generated content (AIGC) has emerged as an innovative educational tool with significant potential. AIGC technologies have the capability to reshape pedagogical practices through various applications, such as virtual patient construction, automated question bank generation, and 3D anatomical simulations [19,29]. These technologies may also help address the uneven distribution of educational resources and facilitate the updating of outdated knowledge. Exploring the application of AIGC in medical education represents both a transformation and expansion of existing teaching models, as well as a forward-looking exploration of future cultivation patterns.

AIME introduces new demands for educational accreditation systems and training models. While traditional medical education accreditation emphasizes faculty strength, physical facilities, and curriculum design, AI enriches these standards by incorporating digital classes, digital resources, and dynamic monitoring. In the context of postgraduate education, AIME necessitates a re-evaluation of training duration, content, and assessment methods. It is crucial for policy makers and practitioners to explore how AI can be reasonably integrated into residents’ learning, ensuring a balance that prevents over-reliance on technology and maintains the importance of clinical practical experience.

### Limitations

This study has several limitations. First, the bibliometric analysis was conducted exclusively using the Web of Science Core Collection database. This singular focus may introduce bias into the results, as it does not account for data from other significant sources. Future research should incorporate additional databases, such as Scopus and Google Scholar, to provide a more comprehensive analysis. Second, while citation frequency is often used as a measure of academic influence, it does not necessarily equate to positive evaluations. Citations can also be made for criticism or rebuttal. Therefore, supplementing bibliometric analysis with content analysis would offer a more accurate reflection of the literature’s true impact. Finally, bibliometric analysis inherently concentrates on published research, which may not capture the most current research trends and developments.

### Conclusions

This study delves into the current landscape of AI applications in medical education, encompassing the geographical distribution of research efforts, recognition of pivotal researchers, identification of key research trends, and exploration of emerging domains. There is a burgeoning interest in AIME and an expanding comprehension of its potential impact. The United States has emerged as a leader in this field, with many institutions standing out as prolific organizations. As the demand for more personalized and effective medical education grows, there is a pressing need for large-scale, rigorously designed studies to provide empirical evidence of AI’s effectiveness and safety.

## Abbreviations

AI: artificial intelligence
AIGC: artificial intelligence–generated content
AIME: artificial intelligence into medical education
DL: deep learning
ML: machine learning

## Data Availability

All data generated or analyzed during this study are included in this published article.
